# Artificial Intelligence-Assisted Drug and Biomarker Discovery for Glioblastoma: A Scoping Review of the Literature

**DOI:** 10.3390/cancers17040571

**Published:** 2025-02-07

**Authors:** Luana Conte, Gerardo Caruso, Anil K. Philip, Federico Cucci, Giorgio De Nunzio, Donato Cascio, Maria Caffo

**Affiliations:** 1 Department of Physics and Chemistry, University of Palermo, 90128 Palermo, Italy; donato.cascio@unipa.it; 2 Laboratory of Advanced Data Analysis for Medicine (ADAM) at DReAM, University of Salento and ASL (Local Health Authority), “V. Fazzi” Hospital, 73100 Lecce, Italy; giorgio.denunzio@unisalento.it; 3 Unit of Neurosurgery, Department of Biomedical and Dental Sciences and Morphofunctional Imaging, University of Messina, 98100 Messina, Italy; gcaruso@unime.it (G.C.); mcaffo@unime.it (M.C.); 4 School of Pharmacy, University of Nizwa, Birkat Al Mouz, Nizwa 616, Oman; philip@unizwa.edu.om; 5 Città di Lecce Hospital, Gruppo Villa Maria, 73100 Lecce, Italy; federico.cucci@puglia.cri.it; 6 Laboratory of Biomedical Physics and Environment, Department of Mathematics and Physics “E. De Giorgi”, University of Salento, 73100 Lecce, Italy

**Keywords:** Artificial Intelligence, Machine Learning, Deep Learning, glioblastoma, biomarker discovery, drug discovery

## Abstract

*Background*: Artificial intelligence (AI) has emerged as a transformative tool in healthcare, particularly in drug and biomarker discovery, where it can enhance precision, streamline discovery processes, and optimize treatment strategies. Despite its potential, the application of AI in glioblastoma (GB) research, especially in identifying novel biomarkers and therapeutic targets, remains underexplored. The aim of this review is to map the existing literature on AI-driven approaches for biomarker and drug discovery in GB, highlighting key trends and gaps in current research. *Design*: Following a PRISMA methodology, this scoping review examined studies published between 2012 and 2024. Searches were conducted across multiple databases, including MEDLINE (PubMed), Scopus, the Cochrane Library, and Web of Science (WOS). Eligible studies were screened, and relevant data were extracted and synthesized to provide a comprehensive overview of AI applications in GB research. *Results*: A total of 224 records were identified, including 210 from PubMed, 104 from Scopus, 4 from WOS, and 6 from the Cochrane Library. After screening and applying eligibility criteria, 33 studies were included in the final review. These studies showcased diverse AI methodologies applied to both drug discovery and biomarker identification, focusing on various aspects of GB biology and treatment. *Conclusions*: This scoping review reveals an increasing interest in AI-driven strategies for biomarker and drug discovery in GB, with promising initial results. However, further large-scale, rigorous studies are needed to validate real-world applications of AI and the development of standardized protocols to enhance reproducibility and clinical translation.

## Simple Summary

Glioblastoma is one of the most aggressive brain tumors, with limited treatment options and poor survival rates. Scientists are now exploring artificial intelligence (AI) to improve drug discovery and identify new biomarkers that can help diagnose the disease earlier and develop better treatments. This study reviews the existing research on how AI is being used in glioblastoma studies, analyzing trends and identifying gaps in current knowledge. By reviewing the scientific literature published over the past decade, we found that AI techniques have been applied in various ways, including predicting drug responses, identifying key genes linked to the disease, and improving personalized treatment approaches. Although the results are promising, AI in glioblastoma research is still in its early stages. More large-scale studies and standardized protocols are needed to ensure these AI-driven discoveries can be used reliably in real-world clinical settings. This research highlights the potential of AI to revolutionize glioblastoma treatment and emphasizes the importance of further investigations to translate these innovations into practical medical applications.

## 1. Introduction

Glioblastoma (GB) is the most aggressive and common primary brain tumor in adults, characterized by its highly invasive nature, resistance to treatment, and poor prognosis [1]. Despite advances in surgical techniques, radiation therapy, and chemotherapy, the median survival for patients with GB remains dismal, typically less than 15 months following diagnosis [2,3]. The complexity of GB lies in its marked intratumoral heterogeneity, rapid growth, and propensity for recurrence, making it an exceptionally difficult cancer to treat effectively.

In 2020, the World Health Organization (WHO-CNS5) updated the classification of central nervous system tumors, with significant changes regarding GB, introducing a more integrated approach, combining morphological and genetic data for a more accurate diagnosis [4]. GB is defined as a wild-type tumor for isocitrate dehydrogenase 1/2 (IDH1/2) genes, characterized by diffuse astrocytic histology and at least one of the following criteria: necrosis, microvascular proliferation, gain of chromosome 7 and loss of chromosome 10, EGFR amplification, and telomerase reverse transcriptase (TERT) gene mutation (4). This new classification, which considers not only histological features but also molecular alterations, reflects advances in the understanding of brain tumor biology.

Standard treatments such as temozolomide (TMZ) and radiotherapy have provided only limited success, with most patients experiencing tumor progression within a few months of therapy [5]. As a result, there is an urgent need for novel therapeutic strategies and more accurate biomarkers that can guide personalized treatment approaches. Traditional methods of biomarker identification and drug discovery have been slow, and the challenges posed by GB’s unique biology have only exacerbated the difficulties in finding effective treatments.

The main genetic alterations characterized in GB include TERT promoter mutation, deletion of the tumor suppressor gene PTEN, high-level gene amplification of the proto-oncogene EGFR, ATRX mutation, and TP53 mutation [4,6,7]. Among these, TERT promoter mutation, PTEN deletion, and EGFR amplification are more frequently present in primary GB (IDH wild-type GB), while ATRX mutation and TP53 mutation are much more common in secondary GB (IDH mutant GB) [7]. Other important genetic alterations reported in GB include NF1, PDGFRA, PIK3R1, PIK3CA, RB1, CDKN2A/B, MDM2, MDM4, CDK4, and H3F3A [8,9,10].

Despite significant advances in the molecular characterization of GB, many challenges remain in implementing these discoveries into clinical management. This is exemplified by the fact that patient survival rates for GB have remained relatively unchanged since 2005, when the Stupp protocol was introduced. The intratumoral heterogeneity and intrinsic molecular complexity of GB, combined with the poor distribution of drugs within the brain due to the natural exclusion provided by the blood–brain barrier (BBB), make effective drug delivery a critical challenge in GB treatment. These findings underscore the need to explore new biomarkers and tools that can help identify novel therapeutic targets for GB.

In this context, innovative approaches, particularly those powered by artificial intelligence (AI), are gaining attention for their potential to overcome these barriers. AI-driven approaches can potentially identify novel drug targets, predict patient responses to therapies, and uncover biomarkers that are critical for personalized medicine [11,12,13,14,15,16]. Recent advancements in AI-powered approaches have significantly transformed the landscape of biomedical research. Machine learning (ML) and deep learning (DL) models enable the rapid analysis of large-scale multi-omics datasets, facilitating the identification of novel biomarkers and drug targets with unprecedented precision. AI-driven computational screening, molecular docking, and predictive modeling have also accelerated the drug discovery process by efficiently prioritizing candidate compounds and repurposing existing drugs for GBM treatment. Furthermore, explainable AI techniques are enhancing our ability to interpret complex biological interactions, paving the way for more personalized and targeted therapeutic strategies. Given the complex and heterogeneous nature of GB, there is an urgent need to synthesize existing research that explores the intersection of AI, drug discovery, and biomarker identification in this field.

This scoping review aims to systematically map the current literature on the application of AI in drug and biomarker discovery for GB, using the keywords “glioblastoma”, “artificial intelligence”, and “drug/biomarker”. The findings from this review will not only provide a comprehensive overview of the current state of knowledge but also guide future research directions, ultimately contributing to the development of more effective and targeted therapies for GB patients.

## 2. Methods

### 2.1. Protocol and Registration

This scoping review was registered on the Open Science Framework (OSF) platform [17].

### 2.2. Eligibility Criteria

This scoping review followed the Population, Concept, Context (PCC) framework to guide the selection of studies (Table 1).

The *population* of interest includes studies specifically focused on patients diagnosed with GB. Studies that examine other types of brain tumors were excluded to maintain the focus on GB. We excluded systematic reviews from this scoping review to focus solely on original research and studies that contribute new data and findings in the areas of biomarker and drug discovery for GB.

The *concept* of this review centers on the application of AI in drug discovery and biomarker identification for GB. This includes studies that utilize AI techniques, such as ML and/or DL, to discover new drug candidates, predict treatment outcomes, or identify novel biomarkers relevant to GB treatment. Studies focusing on AI applications unrelated to GB or those with no direct relevance to drug or biomarker discovery were excluded.

The *context* is limited to research conducted in clinical, translational, or in silico settings, including both retrospective and prospective studies. This review considers a broad range of research environments but excludes studies with no clear pathway to clinical application. Editorials, opinion pieces, and non-peer-reviewed literature were also excluded to ensure that only rigorous, peer-reviewed research was included.

### 2.3. Informational Sources

To identify relevant studies for this review, we conducted a comprehensive search across multiple bibliographic databases, including PubMed, Scopus, WOS, and the Cochrane Library. The search was focused on publications from the last 12 years, specifically between January 2012 and August 2024, to capture the most recent advancements in the field. The initial search strategies were devised by an experienced data specialist (LC) and refined through collaborative discussions among the research team to ensure exhaustive coverage of the topic. Duplicate records were identified and removed to optimize the dataset. Additionally, we manually reviewed the reference lists of key studies and relevant reviews to ensure that no important literature was overlooked. Full details of the final search strategy can be found in the Supplementary Materials.

### 2.4. Search Strategy

The search process employed a combination of specific keywords and phrases, using Boolean operators (AND and OR) to ensure a comprehensive and systematic retrieval of relevant studies (Table 2). The search strategy incorporated both free-text search terms and structured subject headings (e.g., title/abstract terms) where available. Terms related to AI (e.g., “Artificial Intelligence”, “Machine Learning”, and “Deep Learning”) were combined with keywords relevant to drug discovery and biomarker identification (e.g., “Drug” and “Biomarker”) to capture all pertinent literature.

### 2.5. Data Items

We extracted data on key elements from the included studies to provide a detailed overview of the literature. The data items gathered included basic study characteristics such as title, authors, year of publication, methods adopted, data retrieval, algorithms used, founded biomarkers/drugs, and summary of the study. Special focus was placed on the AI applications reported, documenting the types of AI technologies used, such as ML models and DL algorithms involved in drug discovery and biomarker identification.

We also examined the specific pharmacological or therapeutic interventions studied, noting any drug candidates, biomarker discoveries, or therapeutic strategies explored.

Data from the included studies were systematically organized and analyzed to generate a comprehensive synthesis of the evidence: studies were grouped according to their specific AI applications in drug or biomarker discovery for GB. This allowed us to identify recurring themes and variations in AI methodologies.

## 3. Results

A total of 224 records were identified through the database searches, including 110 from PubMed, 104 from Scopus, 4 from WOS, and 6 from the Cochrane Library. No records were identified from other sources or registers. Prior to screening, 24 duplicate records were removed, resulting in 200 unique records for screening.

The titles and abstracts of these 200 records were screened, leading to the exclusion of 116 records that were not relevant to the research question and 51 reports that were excluded for various reasons: book chapter (n = 4); conference paper (n = 4); conference review (n = 1); Editorial (n = 3); Editorial comment (n = 1); Meeting Abstract (n = 1); retracted article (n = 1); review (n = 29); and systematic review (n = 7).

**Figure 1:**
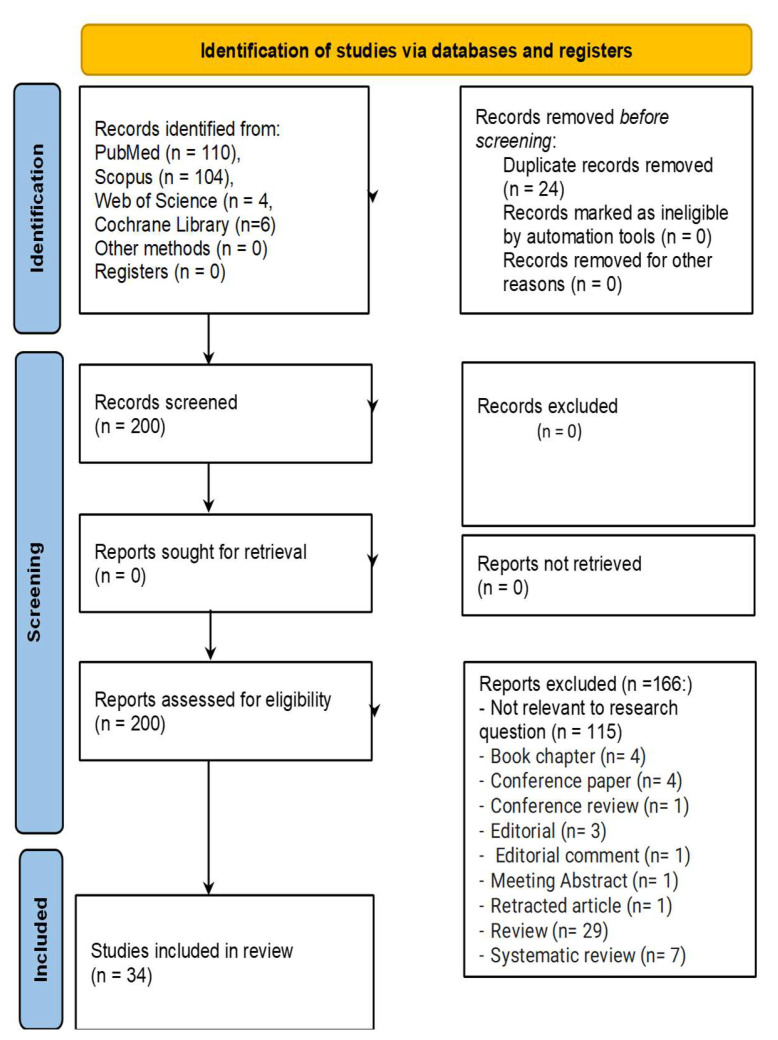
PRISMA 2020 flow diagram [18] of the research.

Ultimately, 33 studies met the inclusion criteria and were included in the final analysis. The process of selecting sources of evidence is detailed in Figure 1, which presents the PRISMA 2020 flow diagram outlining the steps taken from identification to inclusion.

### 3.1. Characteristics of Sources of Evidence

This scoping review includes 33 studies, comprising 33 original research articles, each providing critical insights into the application of AI in biomarker and drug discovery for GB. The literature reviews collectively examine the potential of AI-driven methods in identifying novel therapeutic targets and biomarkers, as well as predicting patient outcomes. These studies span various settings, including in silico, clinical, and translational research environments, and cover different aspects of GB, from biomarker discovery to drug resistance.

### 3.2. Results of Individual Sources of Evidence

The included studies were divided into two categories, presented in separate tables. Table 3 summarizes 21 studies focused on biomarker discovery, while Table 4 covers 12 studies on drug discovery. Each table provides a comprehensive overview of the AI-driven approaches used, along with key findings. Below, we present the results for biomarker discovery, followed by drug discovery in the subsequent section.

**Figure 2:**
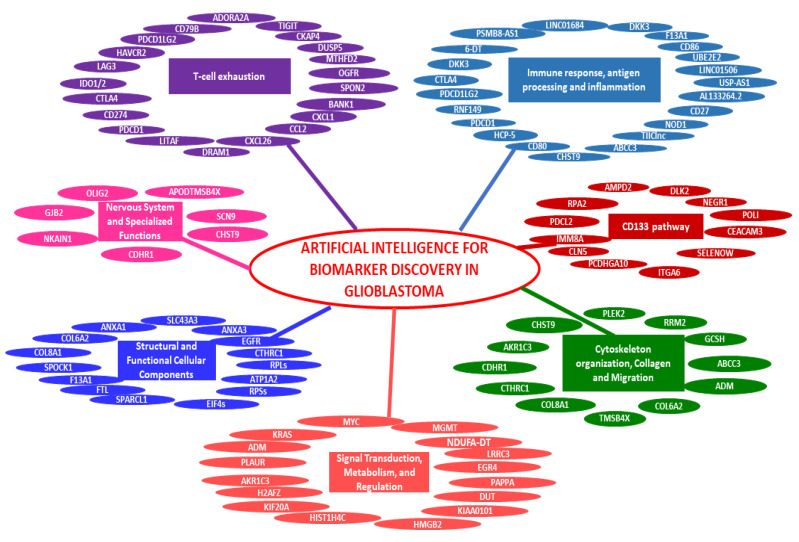
Artificial intelligence for biomarker discovery in glioblastoma: the figure categorizes the main biomarkers identified through AI in the analyzed studies, grouping them into pathways such as T-cell exhaustion, immune response and inflammation, CD133 pathway, signal transduction and metabolism, cytoskeleton organization, and structural and functional cellular components.

**Figure 3:**
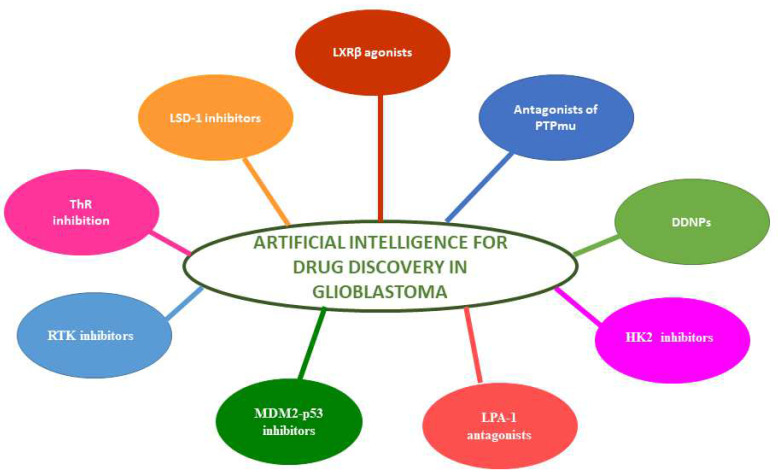
Artificial intelligence for drug discovery in glioblastoma: the figure highlights the main drugs identified in the analyzed studies, including Liver X Receptor beta agonists (LXRβ), Lysine-Specific Histone Demethylase 1 inhibitors (LSD-1), Receptor Tyrosine Phosphatase Mu antagonists (PTPmu), drug-decorated nanoparticles (DDNPs), Hexokinase 2 inhibitors (HK2), Receptor Tyrosine Kinase inhibitors (RTK), Lysophosphatidic Acid Receptor-1 antagonists (LPA-1), Mouse Double Minute 2 and p53 inhibitors (MDM2-p53), and thioredoxin reductase inhibitors (ThR).

Table 4 summarizes 12 studies focused on the use of AI-based models, molecular docking, ML, and high-throughput screening to accelerate the discovery of new drugs or repurpose existing ones for GB treatment. Figure 2 and Figure 3 illustrate the main biomarkers and drugs identified through the use of AI. Figure 2 presents a summary of the main biomarkers identified in the reviewed studies that applied AI-driven methodologies for GB research. The biomarkers are categorized into distinct functional pathways, including immune response and inflammation, T-cell exhaustion, cytoskeleton organization, and metabolic signaling. The studies included in this review utilized various AI techniques, such as ML and DL, to identify these potential biomarkers from transcriptomic, proteomic, and multi-omics datasets. Figure 3 provides an overview of the drugs identified through AI-assisted approaches for GB treatment. The studies reviewed employed computational methods, including molecular docking, virtual screening, and AI-based predictive modeling, to identify both novel compounds and repurposed drugs. The drugs are classified based on their mechanisms of action, including metabolic inhibitors, epigenetic regulators, and signaling pathway modulators.

## 4. Discussion

Gliomagenesis involves numerous biological processes, including the activation of growth factor receptor signaling pathways, the downregulation of apoptotic mechanisms, and an imbalance between proangiogenic and antiangiogenic factors [4]. The identification of novel molecular biomarkers and the development of targeted therapies represent urgent unmet needs that AI-driven approaches have the potential to address.

In this scoping review, we have collected 33 studies, divided into two groups: 21 studies focused on biomarker discovery and 12 studies dedicated to drug discovery. The studies employed various AI-driven techniques, including ML, DL, and multi-omics analysis, to identify novel biomarkers and therapeutic targets associated with prognosis, diagnosis, and therapeutic response.

Several studies have shown that GB presents various types of immune infiltrating cells. The presence of these cellular elements can be predictive of the clinical outcome, and, at the same time, they can represent a valid predictive biomarker. Geo et al. [19] combined the minimum redundancy maximum relevance algorithm (mRMR) and Random Forest (RF) model to identify six immunophenotype-related lncRNAs including USP30-AS1, HCP5, PSMB8-AS1, AL133264.2, LINC01684, and LINC01506. Data obtained using transcriptome data from 144 tumors profiled by The Cancer Genome Atlas (TCGA) based on the single-sample gene set enrichment analysis (ssGSEA) of five immune expression signatures (IFN-g response, macrophages, lymphocyte infiltration, TGF-b response, and wound healing) allowed the recognition of two immunologic profiles (GB immune-high and GB immune-low) closely related to the extent of glial cell infiltration [19]. The immunological characteristics of GB were also evaluated by other authors, who collected 109 immune signatures from public databases (TCGA, GlioVis database, and Wang RNA-seq dataset). Support vector machine–recursive feature elimination (SVM-RFE) was used to evaluate the cases. Finally, to structure an optimal model for the diagnostic prediction of GB subtypes, four ML methods were used, including support vector machine (SVM), RF, extreme gradient boosting (XGBoost), and an artificial neural network (ANN) [27]. In another study, the authors structured a novel computational framework to screen the tumor-infiltrating immune cell-associated lncRNAs (TIIClnc) for developing the TIIClnc signature, analyzing the transcriptome data of purified immune cells, GB cell lines, and bulk GB tissues. Additionally, six ML algorithms were adopted to identify the most interesting TIIClncRNAs [33]. The positive correlation between TIIClnc signature and CD8, PD-1, and PD-L1 was then compared with 95 published signatures in the TCGA and the Xiangya datasets [33].

Cytoskeleton gene WW domain-containing oxidoreductase (WWOX) activity has also been evaluated [32]. This gene seems to be involved in the transcription processes, differentiation, and maintenance. The authors, using RNA-seq data, integrated the use of databases, bioinformatics tools, web-based platforms, and ML algorithms, attempted to identify the role of WWOX-dependent genes. The following genes were identified as relevant WWOX-dependent genes and therefore considered potential biomarkers: PLEK2, RRM2, and GCSH [32].

Tang and collaborators [24], using a supervised ML approach, XGBoost, tried to identify highly predictive genes (>80%) in identifying GB subtypes. The authors, using publicly available GB RNA-sequencing datasets available on the Wang RNA-seq dataset, identified five highly predictive genes: sodium/potassium transporting ATPase interacting (1NKAIN1), ubiquitin-conjugating enzyme E2 (UBE2E2), coagulation factor XIII A chain (F13A1), ring finger protein 149 (RNF149), and Plasminogen activating urokinase receptor (PLAUR), offering potential for diagnostic biomarker development.

The Dickkopf-3 (DKK3) gene shows the ability to inhibit the activation of the WNT pathway implicated in glioma proliferation and invasion. It has been reported that DKK3 expression is essentially absent in GB and significantly reduced in glioma cell lines. These data suggest the role of DKK3 in promoting tumor growth due to its loss of function as a tumor suppression gene [51]. Han and coworkers [23] searched for genes that can interfere with the WNT pathway, including DKK3. Using the brain cancer gene database and an ML system, the authors performed a gene set enrichment analysis (GSEA), a network-based analysis, a survival analysis, and an in vitro drug screening assay based on DKK3 expression. The data showed that high DKK3 expression was negatively correlated with increased antitumor immunity, especially CD8 + and CD4 + T cells, in patients with GB [23].

An interesting study analyzed records from the Rembrandt (REpository for Molecular BRAin Neoplasia DaTa) brain cancer patient-derived dataset. The genomic data were available on the open access Georgetown Database of Cancer (G-DOC) platform and in the NCBI GEO repository as a super series GSE108474. Using GSEA and SVM learning analysis, the authors identified a 33-gene signature that may serve as a biomarker for diagnosis and treatment of GB [31].

Lam et al. [36] developed a liquid chromatography–tandem mass spectrometry (LC-MS/MS)-based proteomic atlas that evaluated the levels of 4794 proteins present in the GB to hallmark histomorphologic niches. The authors therefore carried out a supervised approach to GSEA, in which differential biological programs are defined by different human-annotated sample labels and a region-agnostic approach to explain heterogeneity in GB tumor regions. By using XGBoost models to reveal the functional status of each sample independently, the authors uncover a regionally variable MYC-KRAS molecular axis that interacts with hypoxic phenomena to favor intratumoral heterogeneity. Using multiple independent datasets, the authors also show that tumors over-represented along the KRAS axis show a clinically aggressive and invasive phenotype [36].

An interesting experimental study uses blood-based liquid biopsies primarily focusing on tumor-educated platelets [25]. The authors’ goal was to identify, in GB, new biomarkers and the pathways related to them. Using this method, the authors identified 42 genes and seven pathways (cytoplasmic ribosomal proteins, translation factors, the electron transport chain, ribosome, Huntington’s disease, primary immunodeficiency pathways, and the interferon type I signaling pathway) [25]. Using liquid biopsies, the authors searched for CD133 (a potential biomarker of GB) and proteins associated with it. These data were then compared with 12-month survival through a multi-step ML analysis. The identification of these proteins already involved in various brain functions and in tumor progression mechanisms could prove functional in the search for valid tumor biomarkers [30].

Liu et al. [26] proposed an ML-derived novel gene signature comprising 12 T-cell exhaustion (TEX)-related GBM subtypes. The authors performed a transcriptional profiling analysis of T-cell immunity in the tumor microenvironment of GBM patients. The data allowed the identification of two novel T-cell exhaustions (termed TEX-C1 and TEX-C2). The multi-omics analysis showed distinct immunological, molecular, and clinical characteristics for these two subtypes. Of note, the TEX-C1 subtype presented higher infiltration levels of immune cells, an expressed higher levels of immune checkpoint molecules and a significantly worse prognosis [26]. Recently, it was shown that increased expression of nucleotide-binding oligomerization domain (NOD) 1/2 at NLRC4 was associated with worse survival in GB patients [23]. These biomarkers are members of the NOD-like receptor subfamily C, whose components participate in the regulation of the innate immune response. The authors reached these conclusions using bioinformatics and ML analyses. Nam et al. [37] developed an ML model to study responses to TMZ treatment from combined signatures. The authors therefore examined patient-derived GB stem-like cells, trying to demonstrate the presence of possible mechanisms of resistance to TMZ. The data obtained revealed that the O6-methylguanine-DNA methyltransferase (MGMT) promoter methylation status, hypermutation, and the expression of MGMT, EGR4, ANXA3, PARPA, and LRRC3 can be considered relevant molecular predictors of TMZ response for IDH-wt GBs [37].

In recent experimental research, the authors, using the LASSO algorithm, developed an eight-gene disulfidptosis-based subtype prognostic predictor for GB. Disulfidptosis is a novel cell death mechanism linked to aberrant glucose metabolism and disulfide stress [28]. Huang et al. [29] identified the lncRNA signature NDUFA6-DT as a new potential biomarker in gliomas. The authors found that the values of this biomarker significantly reduced in gliomas, identifying it as an independent protective factor [29].

Zhang and coworkers [29] examined the mechanism of regulated cell death (RCD). The authors studied the RCD landscape, integrating the multi-omics data, including large-scale bulk data, single-cell-level data, glioma cell lines, and proteome-level data, developing an RCD gene pair scoring system. Using a machine learning framework consisting of LASSO, RSF, XgBoost, Enet, CoxBoost, and Boruta, the authors identified seven RCD genes as potential therapeutic targets in glioma, including SLC43A3, which is highly expressed in gliomas.

The ion “permeome” (IP) comprising ion channels, transporters, and other ion-flux-controlling proteins is still poorly studied. The IP gene alterations indicate that ionic flux-mediated bioelectrical signaling via aberrant ion permeome activity is a potential pancancer hallmark. The authors examined 410 survival-associated IP genes in 33 cancer types using an ML approach. The data obtained allowed a clear association between the genes GJB2 and SCN9A and patients’ survival to be identified [38].

Mei et al. [35] evaluated the impact of Oligodendrocyte Transcription Factor 2 (OLIG2) on the survival of GB patients. Collecting radiomic, semantic, and clinical data of each patient, the authors structured an ML model for OLIG2 levels. The data obtained showed that patients with OLIG2 levels equal to or greater than 10% had a worse survival.

In Ye et al.’s [22] study, the authors structured a grade score system potentially able to identify distinct molecular subtypes of glioma. Therefore, mRNA and clinical data from TCGA and CGGA databases were collected. Each grade score was correlated with patients’ clinical molecular pathological features and immune microenvironment. The authors identified KIF20A as a grade score-related biomarker. KIF20A knockdown significantly inhibited tumor growth and invasion and could become a valid biomarker. Similarly, in a recent study [39], the authors, using ML algorithms, attempted to classify cells based on GB scRNA-seq data. The results obtained allowed the following cell groups to be identified, tumor core, tumor periphery, and normal periphery, in binary and multi-class scenarios.

scRNA-sequencing data were used for clustering and an explainable AI framework to find gene biomarkers for the diagnosis and prognosis of recurrent pediatric GB. RF and XGBoost classifiers were constructed to select genes. Finally, five significant genes, namely HMGB2, H2AFZ, HIST1H4C, KIAA0101, and DUT, were screened and in silico-validated through survival analysis and feature plots, and hence proposed as biomarkers for recurring pediatric GB [21].

The drug discovery group demonstrated the use of AI to accelerate the identification of new therapeutic compounds and predict drug efficacy. AI methodologies such as molecular docking, virtual screening, and ML models were employed to discover small-molecule inhibitors, repurpose existing drugs, and explore synergistic drug combinations. Additionally, studies highlighted the application of AI in predicting drug–nanoparticle interactions and targeting specific metabolic or molecular pathways in GB.

The integration of AI in drug discovery for GB presents a paradigm shift in how we approach therapeutic development. Our systematic review of 12 cutting-edge studies reveals diverse applications of AI methodologies, from computational screening platforms such as AtomNet [40,49] to sophisticated ML models and network medicine approaches [41,52]. This technological revolution has accelerated the conventional drug discovery pipeline and enabled rapid identification of novel therapeutic candidates using much fewer resources than would normally be needed in standard drug development processes.

The implementation of AI-powered computational screening, especially through the AtomNet platform [40], has shown great efficiency in the identification of small-molecule antagonists for GB treatment. This approach was further validated through in vitro glioma sphere growth assay testing, showing promising results in reducing tumor growth. The capabilities of the platform were further extended in follow-up studies [49], where it was successfully used in combination with cell-based assays to identify compounds that potentially inhibit glioma cell migration and tumor growth.

Network-based approaches have emerged as powerful tools for understanding complex drug interactions. The application of GCNs [41] has shown effectiveness in predicting synergistic drug combinations, while the combination of advanced network medicine approaches with AI-based models [52] has enabled comprehensive drug response predictions across different glioma subtypes. These methods have enhanced our ability to identify effective drug combinations and predict treatment outcomes.

The integration of molecular modeling and ML approaches [42,43,44] has created new avenues in drug development. Structure-based and ligand-based modeling techniques, coupled with advanced ML algorithms, are enabling the identification of novel therapeutic targets and optimization of drug candidates. These studies have proven the value of integrating traditional approaches in molecular modeling with contemporary AI techniques to increase the efficiency of drug discovery.

Advanced neural network applications, especially Convolutional Neural Networks (CNNs) [47,48], have revolutionized drug response assessment in GB research. These works demonstrate extraordinary success in modeling drug responses in 3D GB neurospheres, thereby providing precious insights into the dynamics of drug–tumor interactions that would otherwise be difficult to obtain with conventional methods. The possibility of evaluating drug efficacy in three-dimensional models has substantially increased our understanding of drug behavior in conditions closer to physiological ones.

Innovation in the field of AI applications has further reached literature mining and drug repurposing through Natural Language Processing (NLP) and high-throughput virtual screening approaches [45]. These methods have proven to be invaluable in the recognition of potential drug candidates from existing compounds and hence speed up the process of drug discovery. A combination of molecular docking with ML [50] has further increased our capability to repurpose existing drugs for GB treatment in a cost-effective approach to drug development.

The studies reviewed employed various AI algorithms in GB drug discovery, each offering distinct advantages and limitations. Traditional ML methods, such as RF, SVM, and XGBoost, have been widely applied for predictive modeling, feature selection, and compound screening. These techniques efficiently process structured datasets, including chemical descriptors and ligand–receptor interactions, enabling the identification of potential drug candidates based on existing pharmacokinetic and pharmacodynamic data. Their main advantage lies in their interpretability, allowing for feature importance analysis and transparent decision-making. However, these models often require manual feature engineering and may struggle with highly complex, non-linear biological relationships.

On the other hand, DL approaches, such as CNNs, DNNs, and GCNs, have demonstrated superior performance in analyzing high-dimensional and unstructured data. CNNs have been particularly useful for imaging-based drug screening, while GCNs have been employed to model molecular interactions and drug–target relationships. These models excel at capturing intricate patterns within large datasets but are often criticized for their black-box nature, limiting interpretability and clinical adoption.

To address these challenges, some studies have integrated ML-based feature selection with DL-driven pattern recognition, creating hybrid AI models that enhance both predictive accuracy and interpretability. For example, RF or XGBoost can be used to identify the most relevant molecular descriptors, which are then processed by DL architectures to refine drug response predictions. Such an approach leverages the advantages of both ML and DL, improving the reliability of AI-driven drug discovery.

Recent advancements have also introduced RL techniques, such as AtomNet^®^, which optimize molecular structures through iterative simulations, enhancing docking scores and bioavailability. Additionally, GANs have been utilized to generate novel molecular compounds, further accelerating the drug discovery pipeline. While these AI-driven methodologies hold great promise, their successful clinical translation will depend on improved model interpretability, validation through experimental studies, and integration with multi-omics data to ensure robust and generalizable predictions.

The clinical implications of such AI-driven approaches are huge. The ability to rapidly screen large compound libraries [40,49], predict responses to drugs [52], and identify synergistic combinations [41] has significantly reduced the time in which drug discovery can be performed. However, translating such computational predictions into clinical success proves to be a challenge. Hence, future research should strive to validate AI predictions with strong clinical trials and develop standardized protocols on how to implement AI in drug discovery.

The studies included in this review employed diverse AI methodologies, ranging from traditional ML models to deep learning-based multi-omics analysis. Despite variations in methodologies, several key trends emerged.

In biomarker discovery, multiple studies identified immune-related biomarkers, particularly those associated with T-cell exhaustion (e.g., PDCD1, CD274, CTLA4, LAG3, and TIGIT), as well as genetic alterations in EGFR, PTEN, and TERT. These findings reinforce the growing recognition of the tumor microenvironment’s role in glioblastoma progression and its potential as a therapeutic target.

In drug discovery, some AI-assisted studies identified promising therapeutic candidates across different approaches. Notably, LXRβ agonists, LSD-1 inhibitors, and RTK inhibitors were frequently highlighted as potential anti-GB agents. Drug repurposing efforts also suggested new applications for existing compounds such as cetirizine and rupatadine (MDM2-p53 inhibitors), which may warrant further experimental validation.

While the AI methodologies varied, a common theme across studies was the ability to identify novel targets that align with previously known GB biology, validating the potential of AI-driven approaches in GB research. Future research should focus on refining AI models to improve prediction accuracy, integrating multi-omics data for a more comprehensive understanding of GB, and prioritizing the experimental validation of newly identified biomarkers and drug candidates.

Based on our analysis, we recommend several strategic directions for future research. First, there should be an effort to establish standardized protocols for AI model validation in GB drug discovery. Second, the integration of computational predictions with experimental validation will definitely make the reliability of AI-driven discoveries stronger. Third, successful approaches, such as AtomNet [40,49] and network medicine [52], shall be expanded to larger patient cohorts to further validate their clinical utility.

In conclusion, the integration of AI in GB drug discovery holds great promise in the development of effective treatments for this complex disease. The diversity of AI applications from computational screening to network medicine testifies to their versatility and potential. Although challenges lie ahead, the continuing development of AI methodologies and their successful applications in identifying promising drug candidates gives hope to the future of AI-driven drug discovery in treating GB.

Together, these two groups of studies provide valuable insights into the potential of AI to transform both biomarker discovery and drug development for GB. The studies offer promising directions for future research and underscore the importance of integrating AI-driven methodologies into GB research to enhance precision medicine approaches and improve patient outcomes [53,54]. To ensure the clinical applicability of AI-driven findings in GB, future research should prioritize prospective clinical trials to validate identified biomarkers and therapeutic targets. Additionally, incorporating more diverse datasets will improve model generalizability across global populations. The successful translation of AI-discovered biomarkers into clinical practice requires standardized validation protocols and regulatory approval. Finally, fostering interdisciplinary collaboration between clinicians and computational scientists will be essential to bridge the gap between AI research and real-world applications.

### Limitations

While this scoping review provides a comprehensive overview of AI applications in biomarker and drug discovery for GB, several limitations should be acknowledged. First, the heterogeneity of study designs, AI methodologies, and outcome measures across the included studies makes it challenging to directly compare results or draw definitive conclusions. The studies also varied in their use of in silico, in vitro, and in vivo models, which may limit the generalizability of findings to clinical practice.

Another limitation lies in the focus on English-language publications, potentially excluding relevant studies published in other languages. Additionally, many of the included studies employed retrospective datasets or in silico models, which may not fully reflect real-world clinical scenarios. The absence of large-scale clinical trials validating AI-driven discoveries limits the direct translation of these findings into clinical applications.

Furthermore, many studies rely on aggregated datasets that may not adequately represent specific subpopulations, such as pediatric and elderly glioblastoma patients, limiting the applicability of AI-driven findings across diverse patient groups. The reliance on predominantly Western datasets also introduces a potential geographic and demographic bias, which could impact the generalizability of the findings.

Additionally, the high computational demands of AI models present another challenge. Many advanced AI techniques require substantial computational resources, which may not be accessible to all research institutions, potentially limiting the widespread adoption of AI-driven approaches.

Another critical issue is the quality of data used to train AI models. Inconsistencies in data annotation, missing values, and variability in imaging and sequencing techniques across different institutions can significantly affect the reliability and reproducibility of AI predictions. Furthermore, the interpretability of AI models remains a major barrier to clinical adoption. Many AI-driven approaches, particularly DL models, operate as “black boxes”, making it difficult for clinicians to understand how predictions are generated. This lack of transparency raises concerns regarding model validation, regulatory approval, and clinical trust. Addressing these challenges will require the development of standardized protocols for data preprocessing, explainable AI frameworks, and interdisciplinary collaborations between clinicians, data scientists, and regulatory bodies.

Finally, the rapid evolution of AI technologies means that some of the AI models and techniques discussed may become outdated as newer, more advanced methods emerge. Future research should address these limitations by incorporating prospective clinical trials, diverse populations, and updated AI methodologies to further validate the potential of AI in GB research.

## 5. Conclusions

This scoping review highlights the transformative potential of AI in GB research, particularly in the areas of biomarker discovery and drug development. The studies reviewed demonstrate that AI-driven approaches, including ML, DL, and multi-omics integration, offer promising pathways for identifying novel biomarkers and therapeutic targets. These advancements are crucial for improving prognosis, guiding personalized treatment strategies, and addressing the challenges posed by the heterogeneity and aggressiveness of GB.

While AI holds significant promise, this review also underscores the need for further validation through large-scale clinical trials and real-world applications. Future research should focus on overcoming existing limitations, such as the reliance on retrospective data and in silico models, and work toward integrating AI into clinical practice to achieve meaningful improvements in patient outcomes.

In conclusion, AI represents a powerful tool for revolutionizing GB treatment and research. Continued investment in AI-driven technologies, along with collaborative efforts between computational scientists and clinicians, will be essential in translating these innovations into tangible benefits for patients facing this devastating disease.

## Figures and Tables

**Table 1:** The Population, Concept and Context (PCC) tool. GB = glioblastoma; AI = Artificial Intelligence; ML = Machine Learning; DL = Deep Learning.

Population	Adult and Pediatric Patients Diagnosed with GB
Concept	Studies examining the application of AI in drug discovery or biomarker identification for GB. Includes AI techniques like ML and DL applied to GB drug or biomarker discovery.
Context	Research conducted in clinical, translational, or in silico settings. Includes a range of healthcare environments, both retrospective and prospective studies.

**Table 2:** Combination of keywords used with the Boolean operator.

Database	Search String
PubMed	((Glioblastoma[Title/Abstract]) AND (Artificial Intelligence[Title/Abstract] OR deep learning[Title/Abstract] OR machine learning[Title/Abstract])) AND (Drug[Title/Abstract] OR Biomarker[Title/Abstract])
The Cochrane Library	(Glioblastoma):ti,ab,kw AND (Artificial intelligence OR deep learning OR machine learning):ti,ab,kw AND (Drug OR Biomarker):ti,ab,kw
Scopus	(TITLE-ABS-KEY (glioblastoma) AND TITLE-ABS-KEY (artificial AND intelligence OR deep AND learning OR machine AND learning) AND TITLE-ABS-KEY (drug OR biomarker))
Web of science (WOS)	((TI = (Glioblastoma)) AND TI = (Artificial intelligence OR deep learning OR machine learning)) AND TI = (Drug OR Biomarker)

**Table 3:** Summary of articles on biomarker discovery for glioblastoma.

No.	Reference	Methods	Data Retrieval	Algorithms Used	Found Biomarkers	Summary of the Study
**1**	[19]	SsGSEA, ML	TCGA	mRMR, RF	USP30-AS1, HCP5, PSMB8-AS1, AL133264.2, LINC01684, LINC01506	Identified six lncRNA biomarkers to distinguish immune phenotypes in GB using TCGA transcriptome data, guiding immunotherapy.
**2**	[20]	GSEA, ML	TCGA	GBM	DKK3	High DKK3 expression was associated with poor prognosis and immunosuppression in GB, providing a therapeutic target.
**3**	[21]	scRNA-Seq, ML	GEO	PCA, KNN, RF, XGBoost, DT	HMGB2, H2AFZ, HIST1H4C, KIAA0101, DUT	Used scRNA-Seq data and ML to identify gene signatures and cell types, improving understanding of recurrent pediatric GB.
**4**	[22]	mRNA data analysis, ML	TCGA, CGGA, GEO	UC, PCA Boruta	KIF20A	Identified KIF20A as a biomarker predicting glioma prognosis and response to TMZ treatment using a novel grade scoring system.
**5**	[23]	Bioinformatics, ML	TCGA	RF	NOD1	High NOD1 expression was associated with poor prognosis and immunosuppression in GB, providing a therapeutic target.
**6**	[24]	ML	WSD	XGBoost	NKAIN1, UBE2E2, F13A1, RNF149, PLAUR	Developed a five-gene classifier to accurately predict GB subtypes, offering potential for diagnostic biomarker development.
**7**	[25]	GSEA, Liquid biopsies, ML	ArrayExpress, TEP	PCA, DT	RPLs, RPSs, ETCs, EIF4s, etc.	Numerous genomic biomarkers were identified from liquid biopsies, revealing different oncogenic pathways involved in GB.
**8**	[26]	Multi-omics, ML	TCGA, GEO, CGGA.	GSEA	CD79B, CKAP4, DUSP5, MTHFD2, OGFR, SPON2, BANK1, CXCL1, CCL2, CXCL26, DRAM1, LITAF	Developed a TEX signature (mRNA, miRNA, and lncRNA) to predict GB prognosis and guide immunotherapy.
**9**	[27]	GSEA, ML	TCGA, GlioVis, WSD	KNN, SVM-RFE, SVM, RF, XGBoost, CMap	PDCD1, CD274, CTLA4, IDO1, IDO2, LAG3, HAVCR2, PDCD1LG2, TIGIT, ADORA2A, VTCN1 , etc.	ML identified GB immune subtypes with different prognoses and immunotherapy responses.
**10**	[28]	SsGSEA, Transcriptomics, ML	TCGA, CGGA	LASSO	CD80, CD86, CTLA4, PDCD1, PDCD1LG2, CD27, etc.	Identified disulfidptosis subtypes in GB, highlighting immunotherapy and targeted therapy implications.
**11**	[29]	SsGSEA, RNA-seq, ML	UCSC Xena, TCGA, Ensembl, GEO, CGGA	GLM, RF, Boruta, GBM, XGBoost, SVM-RFE.	NDUFA6-DT	Identified lncRNA NDUFA6-DT as a prognostic biomarker in gliomas, influencing immune responses and synaptic transmission.
**12**	[30]	ML, proteomics	NIH	LASSO, RFECV	RPA2, AMPD2, DLK2, NEGR1, PDCL2, POLI, CEACAM3, ITGA6, PCDHGA10, SELENOW, IMM8A, CLN5, etc.	Identified serum proteins associated with CD133 using ML to predict 12-month survival in GB patients.
**13**	[31]	GSEA, ML	Rembrandt, G-DOC, GEO	GSEA SVM RBF	ABCC3, ADM, COL6A2, COL8A1, CTHRC1, AKR1C3, CDHR1, CHST9, etc.	Identified a 33-gene signature that may serve as a biomarker for diagnosis and treatment of GB.
**14**	[32]	RNA-seq, ML	TCGA, UCSC	GSEA, SVM-RFE	WWOX-dependent genes: PLEK2, RRM2, GCSH	Identified three key WWOX-dependent genes affecting cytoskeleton reorganization and metabolism in GB.
**15**	[33]	Transcriptomics, ML	TCGA, CGGA, GEO, Xiangya	LASSO, Boruta, XGBoost, SVM, RF, PAMR	TIIClnc signature	Developed a lncRNA signature associated with tumor-infiltrating immune cells to predict prognosis and immunotherapy response in GB
**16**	[34]	Multi-omics, ML	TCGA, CGGA, GEO, GLASS, GTEx, CCLE	LASSO, RSF, XGBoost, Enet, CoxBoost, Boruta	SLC43A3	Developed a gene pair scoring system to predict prognosis and identify therapeutic targets for GB based on RCD phenotypes.
**17**	[35]	Kaplan–Meier, ML	NA	RFE, RF	OLIG2	Developed a predictive model based on OLIG2 expression and MRI features for preoperative prognostic prediction in GB patients.
**18**	[36]	ssGSEA, Proteomics, ML	Recruited patients	XGBoost	KRAS, MYC	Used mass spectrometry and ML to map GB proteome, identifying KRAS and MYC as key drivers of tumor heterogeneity.
**19**	[37]	ML, pharmacogenomics	Recruited patients, TCGA	XGBoost	MGMT, EGR4, PAPPA, LRRC3, ANXA3	Developed an ML model to predict TMZ resistance based on genomic and transcriptomic features in IDH wild-type GB.
**20**	[38]	ML, transcriptomics	TCGA	UC	GJB2, SCN9	Identified ion transport genes such as GJB2 and SCN9A as regulators of GB aggression, providing new drug targets.
**21**	[39]	scRNA-seq, ML	GBTR	PCA, LR, XGBoost, GBM, ET, GNB DT, RF, MLP, LDA, SVM,QDA, KNN, AdaBoost	ATP1A2, SPARCL1, FTL, EGFR, SPOCK1, ANXA1, APODTMSB4X	Applied ML to classify cell groups within GB based on single-cell RNA-seq data, identifying potential biomarkers.

ABCC3 = ATP-Binding Cassette Subfamily C Member 3; AdaBoost = Adaptive Boosting algorithm; ADORA2A = Adenosine A2a Receptor; ADM = Adrenomedullin; AKR1C3 = Aldo-Keto Reductase Family 1 Member C3; AMPD2 = Adenosine Monophosphate Deaminase 2; ANN = artificial neural network; ANXA = Annexin A; APOD = Apolipoprotein D; ATP1A2 = ATPase Na+/K+ Transporting Subunit Alpha 2; BANK1 = B-Cell Scaffold Protein With Ankyrin Repeats 1; CCL2 = C-C Motif Chemokine Ligand 2; CCLE = the Cancer Cell Line Encyclopedia project; CD = Cluster of Differentiation; CDHR1 = Cadherin Related Family Member 1; CEACAM3 = Carcinoembryonic Antigen-Related Cell Adhesion Molecule 3; CGGA = Chinese Glioma Genome Atlas; CHST9 = Carbohydrate Sulfotransferase 9; CKAP4 = Cytoskeleton Associated Protein 4; CLN5 = Ceroid Lipofuscinosis Neuronal 5; COL6A2 = Collagen Type VI Alpha 2 Chain; COL8A1 = Collagen Type VIII Alpha 1 Chain; CMap = connectivity map; CTHRC1 = Collagen Triple Helix Repeat Containing 1; CTLA4 = Cytotoxic T-Lymphocyte Associated Protein 4; CXCL = C-X-C Motif Chemokine Ligand; DKK3 = Dickkopf-3; DLK2 = Delta-Like 2 Homolog; DRAM1 = DNA Damage Regulated Autophagy Modulator 1; DT = Decision Tree; DUSP5 = Dual Specificity Phosphatase 5; DUT = Deoxyuridine 5′-Triphosphate Nucleotidohydrolase; EGFR = Epidermal Growth Factor Receptor; EGR4 = Early Growth Response 4; Enet = Elastic Net; ETC = electron transport chain; F13A1 = Coagulation factor XIII A chain; FTL = Ferritin Light Chain; GB = Glioblastoma; GBJ2 = gap junction; GBM = Gradient Boosting Machine; GBTR = The Gephart Brain Tumor Research Lab in Stanford Neurosurgery; GCSH = Glycine Cleavage System Protein H; G-DOC = Georgetown Database of Cancer; GEO = Gene Expression Omnibus database; GlioVis = Glioma Visualization; GLASS = Glioma Longitudinal AnalySiS; GJB2 = Gap Junction Protein Beta 2; GNB = Gaussian Naive Bayes; GSEA = gene set enrichment analysis; GTEx = The Genotype-Tissue Expression; H2AFZ = Histone 2 A Family Member Z; HAVCR2 = Hepatitis A Virus Cellular Receptor 2; HCP5 = HLA Complex P5; HIST1H4C = Histone Cluster 1 H4 Family Member C; HMGB2 = High Mobility Group Box 2; IDO1 = Indoleamine 2,3-Dioxygenase 1; IDO2 = Indoleamine 2,3-Dioxygenase 2; ITGA6 = Integrin Alpha 6; KIF20A = Kinesin Family Member 20A; KNN = k-nearest neighbors; KRAS = Kirsten Rat Sarcoma Viral Oncogene Homolog; LAG3 = Lymphocyte-Activation Gene 3; LASSO = least absolute shrinkage and selection operator; LDA = Linear Discriminant Analysis; LITAF = Lipopolysaccharide-Induced TNF Factor; LncRNAs = long non-coding RNA biomarkers; LR = Logistic Regression; LRRC3 = Leucine-Rich Repeat Containing 3; MGMT = O6-Methylguanine-DNA Methyltransferase; ML = Machine Learning; MLP = multilayer perceptron; MTHFD2 = Methylenetetrahydrofolate Dehydrogenase 2; mRMR = minimum redundancy maximum relevance algorithm; MYC = MYC Proto-Oncogene, BHLH Transcription Factor; NDUFA6-DT = NDUFA6 Divergent Transcript; NEGR1 = Neuronal Growth Regulator 1; NIH = National Institutes of Health; NKAIN1 = Sodium/Potassium transporting ATPase Interacting 1; NOD1 = nucleotidebinding oligomerization domain 1; OGFR = Opioid Growth Factor Receptor; OLIG2 = Oligodendrocyte transcription factor 2; PAMR = prediction analysis for microarrays; PCA = Principal Component Analysis; PAPPA = Pregnancy-Associated Plasma Protein A; PDCL2 = Phosducin-Like Protein 2; PDCD1 = Programmed Cell Death 1; PDCD1LG2 = Programmed Cell Death 1 Ligand 2; PCDHGA10 = Protocadherin Gamma Subfamily A, Member 10; PD-L1 = Programmed Death-Ligand 1; POLI = DNA Polymerase Iota; PLAUR = Plasminogen activating, urokinase receptor; PLEK2 = Pleckstrin 2; PSMB8-AS1 = Proteasome Subunit Beta 8 Antisense RNA 1; QDA = Quadratic Discriminant Analysis; RBF = radial basis function; RCD = regulated cell death; RFE = recursive feature elimination; RF = Random Forest; Rembrandt = REpository for Molecular BRAin Neoplasia DaTa; RNF149 = Ring finger protein 149; RPA2 = Replication Protein A2; RRM2 = Ribonucleotide Reductase Regulatory Subunit M2; RSF = random survival forest; SCL43A3 = Solute carrier family 43, member 3; SCN9 = Sodium Voltage-Gated Channel Alpha Subunit 9; SELENOW = Selenoprotein W; SLC43A3 = Solute Carrier Family 43 Member 3; SPARCL1 = SPARC-Like Protein 1; SPOCK1 = Sparc/Osteonectin, Cwcv, And Kazal-Like Domains Proteoglycan 1; SPON2 = Spondin 2; ssGSEA = single-sample gene set enrichment analysis; SVM = support vector machine; SVM-RFE = support vector machine–recursive feature elimination; TCGA = The Cancer Genome Atlas; TEPs = tumor-educated platelets; TEX = T-cell exhaustion; TIIClnc = tumor-infiltrating immune cell-associated lncRNAs; TIGIT = T Cell Immunoreceptor with Ig and ITIM domains; TISIDB = tumor–immune system interactions database; TMSB4X = Thymosin Beta 4, X-Linked; TMZ = Temozolomide; UBE2E2 = Ubiquitin conjugating enzyme E2; UC = Unsupervised Clustering; UCSC Xena = University of California Santa Cruz Xena platform; USP30-AS1 = USP30 Antisense RNA 1; VTCN1 = V-Set Domain Containing T-Cell Activation Inhibitor 1; WSD = Wang RNA-seq dataset; WWOX = WW domain-containing oxidoreductase; XGBoost = extreme gradient boosting.

**Table 4:** Summary of articles on drug discovery for glioblastoma.

No.	Reference	Methods	Data Retrieval	Algorithms Used	Founded Drugs	Summary of the Study
**1**	[40]	AI-based computational screening, cell-based assays	Cell cultures	AtomNet^®^	Antagonists of PTPmu	This study uses the AtomNet^®^ platform to identify small-molecule antagonists of PTPmu, targeting GB. Several compounds were tested in glioma sphere growth assays.
**2**	[41]	Network integration	Cell cultures	GCN, DNN, SVM, RF, EN	Anticancer drug combinations	The study applies GCN to predict synergistic anticancer drug combinations, identifying combinations for cancer cell lines, including GB.
**3**	[42]	ML, molecular docking, in vivo testing	ChEMBL BindingDB	SVM, NB	LXRβ agonists	LXRβ agonists were designed through ML and tested in vivo on GB xenograft models, showing promising inhibition of tumor growth.
**4**	[43]	Structure-based and ligand-based molecular modeling, ML	ChEMBL	RF, XGBoost	LSD-1 inhibitors	A combination of molecular modeling and ML was used to discover new LSD-1 inhibitors, tested in GB cells.
**5**	[44]	ML models, in vitro and in vivo testing	ChEMBL	SVM, RF, DNN	4m, 4n	ML models were used to prioritize compounds for anti-glioma properties, leading to in vivo tests in glioma models, with two promising candidates identified that efficiently decreased malignant glioma development in mice, probably by inhibiting thioredoxin reductase activity.
**6**	[45]	AI-based literature mining	QA task, Clinical trials	SAPBERT, NLP	RTK inhibitors, LPA-1 antagonists	NLP was employed to explore medical corpora and clinical trials for potential drug targets for recurrent GB, identifying key drug–gene interactions.
**7**	[46]	High-throughput virtual screening, LDI mass spectrometry	Cell cultures, In vivo model	GLMSD	HK2 inhibitors	Discovery of glycolytic inhibitors for GB, targeting metabolic pathways. Inhibitors were tested both in vitro and in vivo models.
**8**	[47]	Imaging, drug interaction assays	Cell cultures	CNN	Longitudinal drug interaction assessment	This study used CNNs to assess drug synergy in 3D GB neurosphere images over time, revealing dynamic drug interactions.
**9**	[48]	PTML	ChEMBL	KNN, GBM, LDA, LR, DT, RF, XGBoost, GBC, BC, AdaBoost	DDNPs with anti-GB activity	PTML was used to predict drug-nanoparticle complexes targeting GB. Models predicted anti-GB drug interactions with nanoparticles.
**10**	[49]	AI-based AtomNet^®^, cell-based assays	Cell cultures, In vivo models	AtomNet^®^	Antagonists of PTPmu	AI-assisted screening identified small-molecule inhibitors of PTPmu that reduce glioma cell migration and tumor growth in vivo.
**11**	[50]	ML, molecular docking, in vitro assays	ChEMBL	RF, KNN, ET, LGB, HGB, XGBoost, DT, SGD, MLP, AdaBoost	MDM2-p53 inhibitors: cetirizine, rapatadine	This study used ML to repurpose drugs like cetirizine and rupatadine as MDM2-p53 inhibitors, showing anti-GB activity in vitro.
**12**	Munquad 46	Network medicine, AI-based drug response prediction	COSMIC, UCSC Xena, TissueNet	DNN	Drug response prediction	The study developed drug response prediction models using network medicine to identify effective therapies for GB subtypes.

AdaBoost = Adaptive Boosting algorithm; BC = Bagging Classifier; ChEMBL = The European Bioinformatics Institute database; COSMIC = Catalogue of Somatic Mutations in Cancer; CNN = Convolutional Neural Network; DDNPs = drug-decorated nanoparticles; DNN = Deep Neural Network; DT = Decision Tree; ET = Extra Tree; GB = glioblastoma; GBC = Gradient Boosting Classifier; GBM = Gaussian Naive Bayes; GCN = Graph Convolutional Network; GLMSD = graphite dots (GDs)–assisted laser desorption/ionization MS for drug discovery; HGB = histogram-based gradient boosting; HK2 = Hexokinase; KNN = k-nearest neighbors; LDA = Linear Discriminant Analysis; LGB = Light Gradient Boosting; LPA1 = Lysophosphatidic acid receptor-1; LR = Logistic Regression; LSD-1 = Lysine-specific histone demethylase 1; LXRs = Liver X Receptors; MLP = multilayer perceptron; NB = Naive Bayes; NLP = Natural Language Processing; PTML = perturbation theory machine learning; PTPmu = receptor tyrosine phosphatase mu-dependent adhesion; QA = question/answering; RF = Random Forest; RTK = receptor tyrosine kinase; SAPBERT = self-aligning pretrained Bidirectional Encoder Representations from Transformers; SGD = stochastic gradient descent; SVM = support vector machine; XGBoost = extreme gradient boosting.

## Supplementary Materials

The following supporting information can be downloaded at: https://www.mdpi.com/article/10.3390/cancers17040571/s1, PRISMA 2020 Checklist.
